# Engineering hybrid exosomes by membrane fusion with liposomes

**DOI:** 10.1038/srep21933

**Published:** 2016-02-25

**Authors:** Yuko T. Sato, Kaori Umezaki, Shinichi Sawada, Sada-atsu Mukai, Yoshihiro Sasaki, Naozumi Harada, Hiroshi Shiku, Kazunari Akiyoshi

**Affiliations:** 1 JST-ERATO, Akiyoshi Bio-nanotransporter Project, Kyoto University Katsura, Nishikyo-ku, Kyoto 615-8510, Japan; 2 Department of Polymer Chemistry, Graduate School of Engineering, Kyoto University Katsura, Nishikyo-ku, Kyoto 615-8510, Japan; 3 Department of Immuno-Gene Therapy, Graduate School of Medicine, Mie University, Tsu 514-8507, Japan

## Abstract

Exosomes are a valuable biomaterial for the development of novel nanocarriers as functionally advanced drug delivery systems. To control and modify the performance of exosomal nanocarriers, we developed hybrid exosomes by fusing their membranes with liposomes using the freeze–thaw method. Exosomes embedded with a specific membrane protein isolated from genetically modified cells were fused with various liposomes, confirming that membrane engineering methods can be combined with genetic modification techniques. Cellular uptake studies performed using the hybrid exosomes revealed that the interactions between the developed exosomes and cells could be modified by changing the lipid composition or the properties of the exogenous lipids. These results suggest that the membrane-engineering approach reported here offers a new strategy for developing rationally designed exosomes as hybrid nanocarriers for use in advanced drug delivery systems.

Biological cells secrete a variety of membrane-derived vesicles. These extracellular vesicles (EVs) are involved in relatively long-range intercellular communication. Recent studies have shown that exosomes shuttle nucleic acids (mRNA or miRNA) between cells and regulate the function of the recipient cell at a post-transcriptional level, and have fundamentally changed our understanding of gene regulation^1^,^2^,^3^.

Exosomes are small membrane vesicles with a diameter of 50–200 nm that are formed in endosomal multivesicular compartments and secreted when they fuse with the plasma membrane. After their secretion, the lipid bilayer of the exosome protects its cargo from plasma and immune components and helps to deliver the cargo to recipient cells by endocytosis or fusion without compromising the intrinsic function of the cargo^4^,^5^,^6^. Exosomes can be loaded with a diverse range of biological molecules, including cytosolic proteins, membrane receptors, and nucleic acids.

Because of these delivery functions, the exosomes have been investigated to use as novel nanocarriers for advanced drug delivery systems. Many researchers have successfully packaged a cargo of interest into exosomes using a variety of methods^7^,^8^. One of the most widely used approaches for loading therapeutic agents into exosomes involves transfecting exosome-producing cells, inducing them to overexpress a specific gene product^9^,^10^,^11^,^12^. By genetic modification of the parental cell, it has been possible to introduce a variety of biological molecules, such as miRNAs, water-soluble proteins, and cell surface proteins, into exosomes^9^,^10^. The exosomal surface can be also modified with specific membrane proteins using similar techniques^11^,^12^. On the other hand, non-genetic methods were reported to afford exosomes loading hydrophilic and/or hydrophobic cargos which are directly introduced to exosome-generating cells by various cellular uptake techniques^7^,^13^.

Direct loading of isolated exosomes by incubating them with the chosen cargo has also been reported^7^,^8^. Hydrophilic miRNAs as well as relatively hydrophobic molecules, such as steroids (e.g., cucurbitacin), polyphenols (e.g., curcumin), and anticancer agents (e.g., doxorubicin) have been introduced into exosomes by simply mixing the cargo with the exosomes. Transfection using cationic lipids^14^ and electroporation^15^,^16^,^17^,^18^, which applies an electric field to a suspension containing the exosomes and the intended cargo, have also been used to load cargoes, particularly siRNAs, into exosomes. However, such techniques may have adverse effects on the exosomes and cargoes, by promoting aggregation for example^19^.

For other applications of exosomal carriers in drug delivery systems, it may become necessary to modify and tune the exosomal interface (i.e., lipid bilayer membrane). Here, we investigated a novel and facile membrane-engineering strategy to modify the surface of exosomes using direct membrane fusion between synthetic liposomes and exosomes post secretion from parent cells. This method allows us to optimize the properties of the exosome surface in order to decrease its immunogenicity and increase its colloidal stability, and improve the half-life of exosomes in blood. However, the hydrophobic properties of lipid molecules hinder direct loading of lipids into exosomes. Genetic modification of the exosomes’ lipid membrane is also difficult because lipid biosynthesis involves a large number of proteins, and the process by which lipids are sorted from the host cell to the exosome is not clearly understood. In this context, we proposed a new approach to prepare engineered hybrid exosomes by fusing the membranes of exosomes and liposomes using the freeze–thaw method (Fig. 1). The properties of the exosome surface can be easily modified using liposomes embedded with peptides or antibodies as targeting moieties or poly(ethylene glycol) (PEG)^20^,^21^. To provide a proof-of-concept for engineering exosome using this membrane fusion approach, we prepared exosomes in cells expressing the tyrosine kinase receptor HER2^22^, and these HER2-containing exosomes were fused with phospholipid liposomes. In addition to evaluating the membrane fusion of exosomes with liposomes, we performed flow cytometry and fluorescence microscopy to examine the performance of the obtained exosomes as nanocarriers.

## Results

### Interaction of Raw 264.7 cell-derived exosomes and liposomes

**Figure 2:**
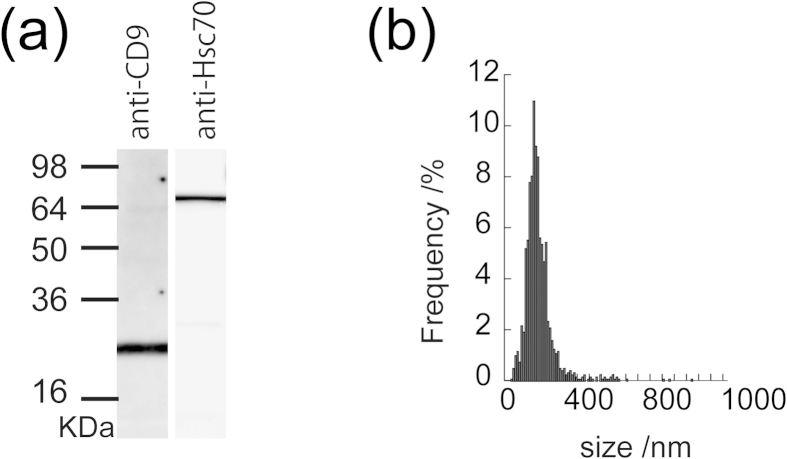
Characterization of Raw 264.7 exosomes. (**a**) Western blot analysis of exosomes using anti-CD9 or anti-Hsc70 antibodies. (**b**) Size distribution profile, as determined by nanoparticle tracking analysis.

Exosomes were isolated from the supernatants of Raw 264.7 cell cultures by differential centrifugation and micro-filtration. Western blot analysis of Raw 264.7 cell-derived exosomes revealed high expression of exosome markers, especially heat-shock proteins (e.g. Hsc70) and tetraspanin (CD9) (Fig. 2a). Nanoparticle tracking analysis (NTA) revealed that the hydrodynamic diameter of the exosomes was 150 ± 56 nm (mean ± standard deviation) (Fig. 2b).

The Raw264.7 cell-derived exosomes were fused with liposomes by the freeze–thaw method. Fluorescently labelled liposomes were prepared by extruding an aqueous liposome dispersion comprising 1,2-dioleoyl-*sn*-glycero-3-phosphocholine (DOPC; zwitterionic), 1,2-dioleoyl-*sn*-glycero-3-phospho-l-serine; (DOPS, anionic), 1,2-dioleoyl-3-trimethylammonium propane (DOTAP; cationic) or 1,2-distearoyl-*sn*-glycero-3-phosphoethanolamine-*N*–[methoxy(polyethylene glycol)-2000] (PEG-DSPE; Mw 2000 PEG was introduced to lipid head) in the presence of fluorescently-lebelled lipids, 1,2-dimyristoyl-*sn*-glycero-3-phosphoethanolamine-*N*-(lissamine rhodamine B sulfonyl) (rho-DMPE) or 1,2-dimyristoyl-*sn*-glycero-3-phosphoethanolamine-*N*-(7-nitro-2-1,3-benzoxadiazol-4-yl) (NBD-DMPE) (both 1 mol%). The Raw 264.7 cell-derived exosomes were mixed with each of the labelled liposomes at a volumetric ratio of 1:1. To induce membrane fusion, the mixtures were frozen in liquid nitrogen and thawed at room temperature for 15 min.

**Figure 3:**
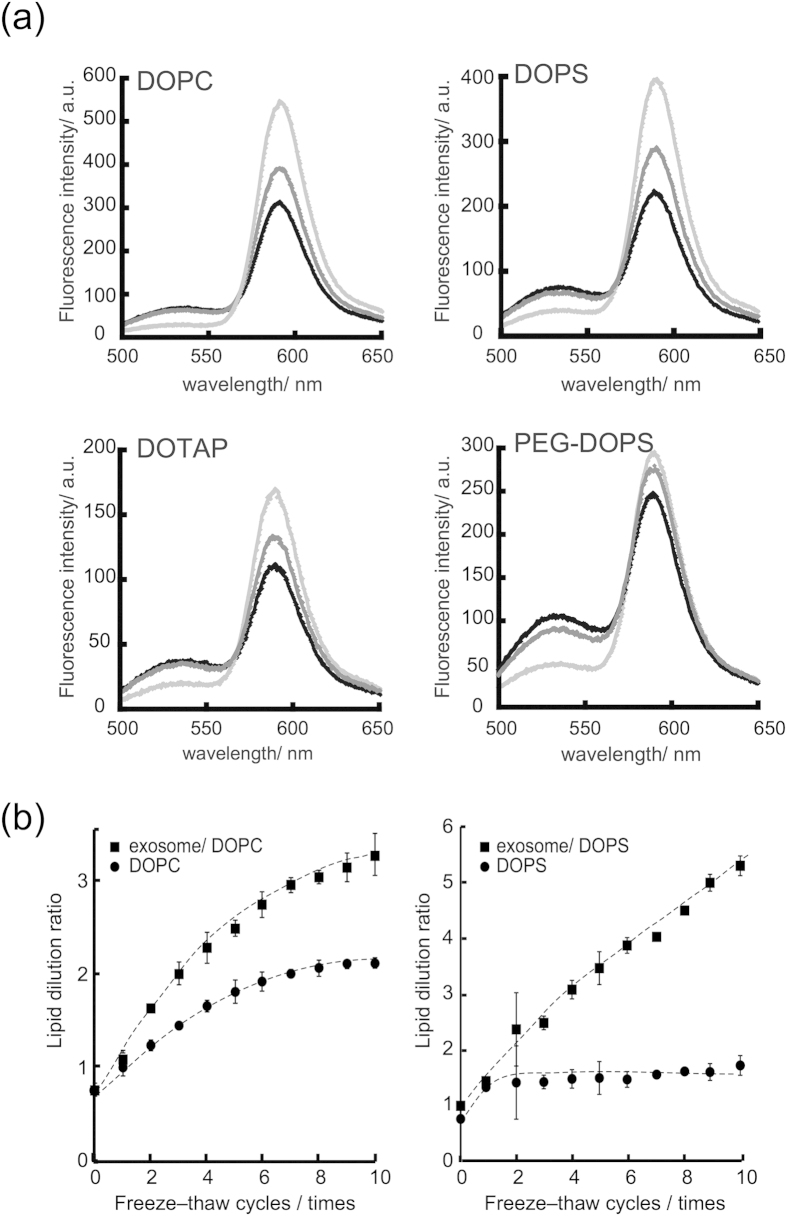
Interaction of Raw 264.7 exosomes with liposomes containing various lipids. (**a**) Fluorescence spectra with excitation at 460 nm. The light grey, dark gray and black lines represent the spectra before, 5 times and 10 times after freeze thawing, respectively. (**b**) Relationship between the number of freeze–thaw cycles and the dilution ratios of DOPC liposomes or exosome–DOPC liposomes (left), the dilution ratios of DOPS liposomes or exosome–DOPS (right).

The efficiency of exosome and liposome fusion was quantitatively evaluated in terms of the lipid mixing ratio measured using a fluorescence resonance energy transfer (FRET) assay using a set of NBD and rhodamine-labelled lipid, which is widely used for the analysis of liposome–liposome, liposome–cell, and virus–cell fusion^23^,^24^,^25^,^26^. For example, when NBD-DMPE is excited at a wavelength of 460 nm, the resulting solutions emit fluorescence at 530 and 588 nm, reflecting the emission from NBD-DMPE and rho-DMPE, respectively. After subjecting the exosome–liposome mixture to one or more freeze–thaw cycles, the fluorescence intensity at 530 nm increased and the intensity at 588 nm decreased as the number of cycles was increased (Fig. 3a). These results demonstrate that the fluorescent lipids in the liposome were diluted by lipid molecules or other membrane components (e.g., membrane proteins) present in the exosome. The lipid dilution ratio as fusion efficiency was evaluated using a calibration set of fluorescence spectra. Figure 3b shows the relationship between the efficiency and the number of freeze–thaw cycles.

When unlabelled DOPC liposomes were interacted with fluorescently labelled liposomes, the lipid dilution ratio was almost 2.1 ± 0.1 after 10 freeze–thaw cycles, whereas, the efficiency for the exosome–liposome system was 3.3 ± 0.2, which was higher than that of the liposome–liposome system. As for the mechanism to explain the difference of the fusion efficiencies, interaction of liposomes with the protein existed in exosomal membrane or glycosylation is considered to be a plausible factor. The efficiency of each liposomal system is presented in Table 1, which shows relatively high fusion efficiencies for all of the liposomal systems used in this study, irrespective of the type of lipid. Although the diameter changes of exosomes after fusion with liposomes were evaluated by nanoparticle tracking analysis, it was difficult to detect statistically the differences of the averaged sizes and the size distribution after the freeze thawing (Supplementary Table S1 for averaged size and Figure S1 for size distribution). The morphological change of exosome after the freeze thawing was also examined by negatively stained transmission electron microscopy (TEM). Though TEM study could not represent averaged morphology, there were not any apparent morphological difference between the exosomes before and after membrane fusion by the freeze thawing treatment (Supplementary Figure S2).

### Isolation of exosomes from HER2-expressing CMS7 cells

**Figure 4:**
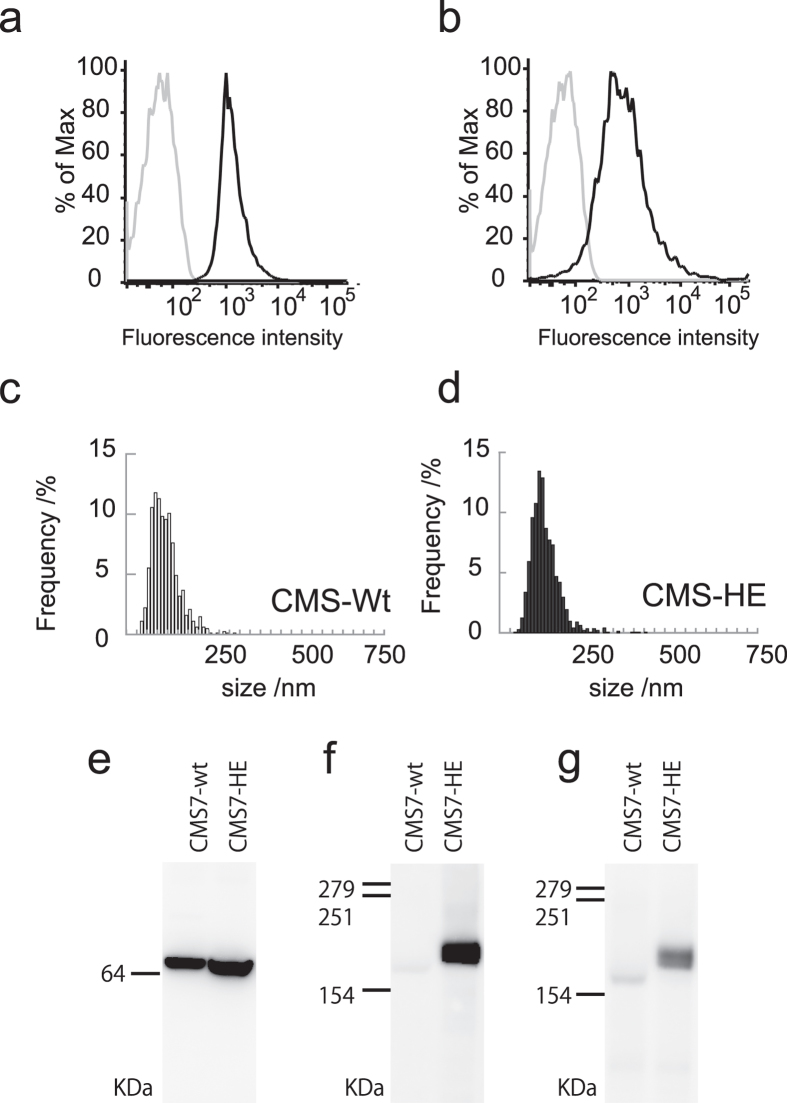
Characterization of CMS7 exosomes. (**a,b**) FACS analysis of CD63 expression on the surface of exosomes from CMS7-wt (**a**) and CMS7-HE (**b**) cells. The grey and black lines represent isotype controls and anti-CD63, respectively. (**c,d**) Size distribution profile, as determined by nanoparticle tracking analysis, of exosomes from CMS7-wt (**c**) and CMS7-HE (**d**) cells. (**e–g**) Western blot analysis of exosomes from CMS7-wt or CMS7-HE cells using anti-Hsc70 (**e**), anti-HER2 (**f**), or anti-phosphorylated HER2 (**g**) antibodies.

To further examine the applicability of the membrane fusion technique used here, we investigated the fusion behaviour between exosomes bearing a specific membrane protein and the liposomes. Exosomes were isolated from the culture supernatants of wild-type CMS7 cells (CMS7-wt cells) and genetically modified CMS7 cells overexpressing the human HER2 receptor (CMS7-HE cells)^27^,^28^. The surface protein CD63, a representative exosome marker, was detected in exosomes derived from both cell types by fluorescence-activated cell sorting analysis using an exosome bead-coupling technique (Fig. 4a,b). The hydrodynamic diameters of exosomes derived from CMS7-wt and CMS7-HE, as measured by NTA, were similar (Fig. 4c,d).

The loading of HER2 protein on the exosome membrane was evaluated by western blot analysis using anti-Hsc70 as an exosome marker and anti-HER2. As expected, HER2 was only detected in CMS7-HE–derived exosomes as a predicted band (Fig. 4f), whereas Hsc70 was detected in exosomes from both cell types (Fig. 4e). Furthermore, HER2 activity was determined using a corresponding anti-phospho antibody (Fig. 4g), which confirmed that HER2 was activated by auto-phosphorylation of tyrosine residues. The presence of phosphorylated HER2 indicates that the exosome carried active HER2 protein. The protein concentration of HER2 on exosomes was also evaluated using a standard curve prepared using samples containing known concentrations of HER2. These studies revealed that the obtained exosomes contained about 2–3.3 ng of HER2 per 1 μg of total protein.

### Interaction of genetically modified exosomes embedded with HER2 protein and liposomes

Table 2 shows the fusion efficiency for each lipid system. As expected, significant membrane fusion was observed for all of the lipid systems evaluated here. These efficiencies of CMS7-HE were relatively higher than that of Raw 264.7 exosomes system. We do not have clear answer for the difference of fusion efficiency at this moment. Membrane component of exosome (e.g. lipid composition, membrane protein content) were significantly different depending on its origin so that the difference would alter the interaction between liposome and exosome related to fusion efficiency.

**Figure 5:**
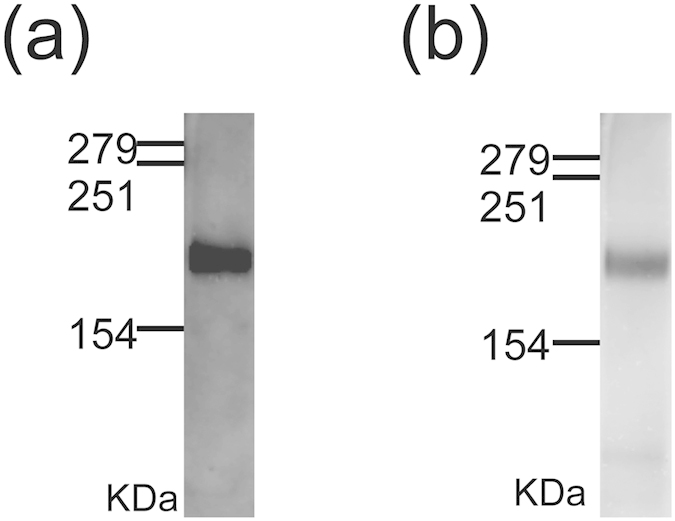
Western blot analysis of CMS7-HE exosomes following membrane fusion with DOTAP liposomes. Western blotting was conducted using anti-HER2 (**a**) or anti-phosphorylated HER2 (**b**) antibodies.

Next, we performed western blotting to evaluate the properties of exosomes embedded with HER2 after the freeze–thaw cycles with liposomes. HER2 and phosphorylated HER2 were detected in the exosome–liposome mixtures (Fig. 5a,b), as in untreated exosomes (Fig. 4f,g). These results indicate that genetic modification of exosomes and fusion-based membrane modification techniques can be combined, and that exosome–liposome hybrids carrying specific proteins can be obtained by freeze–thaw methods.

### Cellular uptake of membrane-engineered exosomes

Exosomes have valuable delivery functions, which can be exploited in the development of novel nanocarriers for advanced drug delivery. In general, exosomes deliver their molecular cargo to the cytosol via endocytosis or membrane fusion^4^,^5^,^6^,^29^. Therefore, we determined the cellular uptake efficiency of the membrane-engineered exosomes.

**Figure 6:**
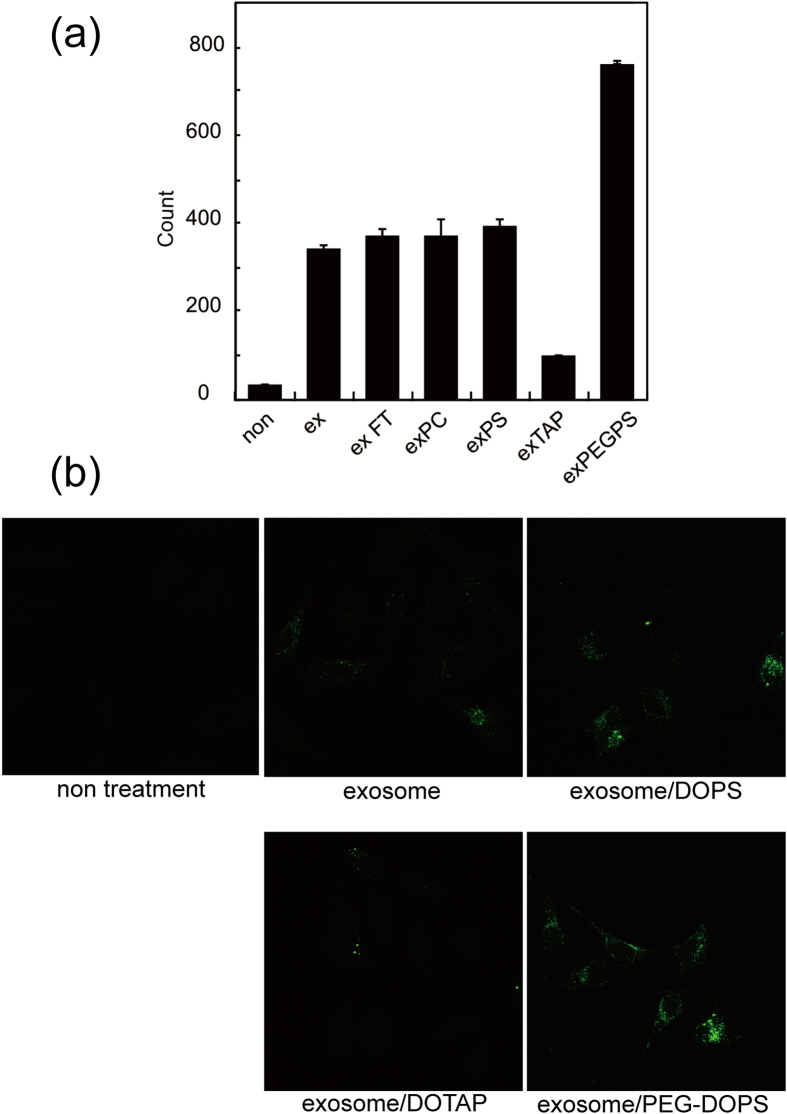
Cellular uptake of exosomes or exosome–liposome hybrids. (**a**) Mean fluorescence of cells, as measured by flow cytometry. (**b**) Confocal laser scanning microscopy images of HeLa cells incubated with exosomes or exosome–liposome hybrid. Green fluorescence represents 5- or 6-(*N*-succinimidyloxycarbonyl)-fluorescein 3′,6′-diacetate-labelled exosome.

HeLa cells were incubated with 5- or 6-(*N*-succinimidyloxycarbonyl)-fluorescein 3′,6′-diacetate (CFSE)-labelled exosomes or exosome–liposome hybrids for 4 h and cellular uptake was quantified by FACS analysis and observed by confocal laser scanning microscopy. Prior to the cellular uptake experiment, it was confirmed that the labeling efficiency did not significantly change in the various systems. In Fig. 6, the cellular uptake profile was similar between DOPC–exosome hybrids and DOPS–exosome hybrids, which indicates that neutral and anionic lipids did not affect the cellular uptake of these exosomes. However, incorporation of a cationic lipid (i.e., DOTAP) into the hybrid exosome decreased the cellular uptake efficiency. On the other hand, the cellular uptake of the hybrid exosomes with PEG-DOPS was significantly increased, by almost two-fold, compared with the unmodified exosomes. To rule out the possibility that the positive effect of PEG-DOPS is attributed to a fraction of the unfused PEG-DOPS liposomes and exosomes but not the hybrid exosomes cellular uptakes with or without freeze-thaw cycles were compared by using FACS under the same condition. The uptake of the hybrid exosomes with PEG-DOPS treated by freeze-thaw process was significantly increased more than that of the mixture of exosome and DOPS without freeze-thaw, showing the increased cellular uptake was attributed to the hybridization of the exosomes (Supplementary Figure S3). These results indicate that the interactions between exosomes and cells can be modified by introducing exogenous lipids to hybrid exosomes.

## Discussion

In this study, we developed engineered hybrid exosomes by fusing the membranes of exosomes with liposomes using the freeze–thaw method. The freeze–thaw method^30^,^31^,^32^ is a relatively simple physicochemical process that was selected to prepare the hybrid exosomes by membrane fusion. The freeze-thaw method has been used to convert multi-lamellar liposomes to large uni-lamellar liposomes or incorporate water-soluble molecules into the internal water phase of liposomes, which suggests that this method disrupts plasma membranes by temporary formation of ice crystals^33^,^34^,^35^. Accordingly removing the water molecules from the hydrophilic surface of the lipid bilayer membrane by freezing appears to cause significant structural and functional changes, including hydration-dependent phase changes, lateral phase separation of membrane components, and membrane fusion; the latter may occur as a result of membrane reconstitution^36^,^37^. These findings prompted us to use the freeze–thaw method to induce fusion or mixing of exosome and liposome membranes. In addition to being simple, this physical technique avoids contaminating the membranes with unwanted chemicals such as calcium or PEG, for example, which are used in other chemical fusion methods. Exosomes embedded with a specific membrane protein (in this case HER2) were also fused with various liposomes at relatively high efficiencies, confirming that membrane engineering can be combined with genetic modification of exosomes.

Cellular uptake studies of the hybrid exosomes confirmed that the delivery function of the exosomes could be modified by changing the lipid composition or the properties of the exosome by membrane fusion. Although it is unclear why the cellular uptake of cationic DOTAP–exosome hybrids was reduced, it is possible that cationic lipids might influence the proteins involved in the interactions between the exosomes and cells for cellular uptake. On the other hand, the cellular uptake of the PEG-modified exosomes was significantly increased. Generally, the presence of PEG on the liposome surface increases their circulation time in blood while reducing their uptake by mononuclear phagocytes (namely, stealth liposomes)^38^. These findings indicate that PEG reduces interactions between the liposomes and cellular membranes for effective cellular uptake of the PEG-modified exosomes. One of the possible explanations for the unexpectedly high uptake efficiency is that PEG existed on exosomal surface reduced electrostatic repulsions of anionic exosome surface with anionic cell surface to increase the exosome-cell interaction for increased cellular uptake. Another possibility is PEG-induced membrane fusion^39^ which is caused by small perturbations in lipid packing within the bilayer leaflets contacted by the liposomes.

Recently, it was reported that exosomes transport not only water-soluble cargoes like proteins and RNA, but also lipophilic molecules^13^. Therefore, exosomes can carry bioactive lipids such as lipid signals or mediators between proximal or distal cells, and their fate is at least partly regulated by these lipids. Furthermore, exogenous membrane proteins can be incorporated into exosomes using this engineering method. Recently, we reported a new one-step method for preparing proteoliposome by cell-free membrane protein synthesis in the presence of liposomes. For example, liposomes containing connexin were developed using this method, and were capable of delivering a hydrophilic oligo-peptide to cells via connexin-mediated communication through gap junctions between the proteoliposome and cells^40^. Fusion of the proteoliposome and exosome membranes facilitated the incorporation of the membrane protein into the exosomes. The exosome–liposome fusion method should also be useful for loading therapeutic agents into exosomes, a field that is now being researched by our group.

In conclusion, the present results suggest that the membrane-engineering method used here represents a new strategy to yield hybrid exosomes as novel biological nanotransporters (bio-nanotransporter). These engineered hybrid exosomes can be used to transport exogenous hydrophobic lipids incorporated *in vitro* by the membrane fusion method to recipient cells as well as hydrophilic cargoes within the exosomes as advanced drug delivery systems.

## Methods

### Materials

DOPC, DOPS, and PEG-DSPE, were purchased from NOF Corp. Ltd. (Tokyo, Japan). DOTAP (chloride salt), NBD-DMPE, and rho-DMPE were purchased from Avanti Polar Lipids (Alabaster, AL, USA). Anti-Hsc70 (Anti-Hsc70 rat monoclonal antibody [1B5]), anti-HER2 (anti-ErB2), anti-CD9, anti-phosphorylated HER2 (p-Neu[Tyr1248]), phycoerythrin (PE)-conjugated mouse anti-human CD63, horseradish peroxidase (HRP)-conjugated goat-anti rabbit-IgG, and PE-conjugated mouse IgG1 were purchased from Enzo Life Sciences (Farmingdale, NY, USA), abcam (Cambridge, UK), Santa Cruz Biotechnology (Santa Cruz, CA, USA), Biolegend (San Diego, CA, USA), and BD Bioscience (San Jose, CA, USA), respectively. ECL Western Blotting Detection Reagent and PD SpinTrap G-25 were purchased from GE Healthcare Japan (Tokyo, Japan). Aldehyde/sulfate latex beads (4% w/v, 4 μm) and stain buffer (FBS) were purchased from Life Technologies (Carlsbad, CA, USA) and BD Bioscience (CA, USA), respectively. CFSE was obtained from Dojindo Molecular Technologies, Inc. (Kumamoto, Japan).

### Cell culture

Mouse fibroblast sarcoma-derived CMS7-wt, CMS7-HE, and Raw 264.7 macrophages were kindly provided by Professor Shiku (Mie University, Japan). HeLa cells were purchased from JCRB Bank (Japanese collection of Research Biosources 9004, Osaka, Japan) Raw 264.7 cells were cultured in Dulbecco’s Modified Eagle Medium while CMS7-wt and CMS7-HE cells were cultured in RPMI 1640 medium supplemented with 10% fetal bovine serum (FBS) and 1% penicillin-streptomycin (PS). HeLa cells were cultured in MEM medium with 1 % Non-essential amino acids solution and 10 % FBS and 1% PS. All cell lines were cultured in a 5% CO_2_ incubator at 37 °C.

### Exosome isolation

Exosomes were isolated by differential centrifugation and micro-filtration as previously described^10^,^41^,^42^. Briefly, the cells (Raw 264.7, CMS7-wt, and CMS7-HE) were incubated for 15 h in exosome-free FBS, which had been centrifuged at 100,000 × *g* before use. The culture supernatants obtained from each cell culture were collected, centrifuged at 300 × *g* for 10 min to remove cells, at 1,200 × *g* for 20 min, and finally at 12,000 × *g* for 30 min (all at 4 °C), followed by filtration through a 0.22-μm filter. Exosomes were pelleted by ultracentrifugation at 120,000 × *g* for 70 min at 4 °C. The pellets were rinsed with phosphate buffered saline (PBS), ultracentrifuged again, and dispersed in PBS. The protein concentrations of the exosome suspensions were determined using BCA assays (Thermo Scientific, Waltham, MA, USA) and were adjusted to 300 μg/mL before use.

### Nanoparticle tracking analysis

The size distribution of the exosomes and liposomes were analysed using a Nano Sight LM10 instrument Marvern instruments Ltd, Worcestershire, UK) equipped with NTA software version 2.3. The particle suspensions were diluted with PBS to a concentration of 1–8 × 10^8^ particles/mL for analysis.

### Western blot analysis

Exosomes were suspended in sample buffer (50 mM Tris-HCl pH 6.8, 1% sodium dodecyl sulphate (SDS), 0.05% bromophenol blue, 5% glycerol, 50 mM dithiothreitol, 15 mM Na_3_PO_4_, 0.5 mM (phenylmethanesulfonyl fluoride) and heated at 70 °C for 15 min. Exosomes were run on SDS–polyacrylamide gels, transferred to hydrophobic polyvinylidene difluoride (PVDF) membranes, and blocked with 0.5% skimmed milk or 1% bovine serum albumin buffer. The blots were incubated with primary antibodies to CD9, Hsc70, HER2, or phosphorylated HER2 at room temperature for 10 min (CD9 and Hsc70) or at 4 °C overnight (HER2 and phosphorylated HER2). After washing, the membranes were probed with a secondary HRP-conjugated goat anti-rabbit IgG antibody. Signals were detected using ECL western blotting detection reagent.

### Liposome preparation

Liposomes were prepared using an established protocol^43^. Briefly, an appropriate amount of phospholipid and fluorescently labelled lipid (NBD-DMPE and rho-DMPE) were dissolved in 150 μl of organic solvent (chloroform:methanol = 2:1, 30 mM for DOPC, 10 mM for DOTAP, 1 mol% NBD-DMPE and 1 mol% rho-DMPE). The solvent was evaporated under argon gas flow and the residual trace solvent was completely removed *in vacuo* to yield a thin film on the wall of a glass vial. The lipid film was hydrated by adding 150 μl of PBS buffer and vortexing the tube at a temperature above the phase transition temperature. The lipid suspension was extruded through a polycarbonate filter with 100-nm pores. The total lipid concentrations of the liposomes were adjusted to 100 μM.

### Membrane fusion

The exosome and liposome membranes were fused using an established freeze–thaw method^30^,^31^,^32^. Exosomes were mixed with 100 μM liposomes labelled with 1 mol% NBD-DMPE and rho-DMPE (1:1 by volume). The mixtures were frozen in liquid nitrogen and thawed at room temperature for 15 min. The freeze–thaw cycle was repeated for several times. The fusion efficiency was evaluated using established FRET assays^30^,^31^,^32^. Briefly, fusion was monitored using a fluorescence couple of NBD-DMPE and rho-DMPE within the lipid bilayer membrane. The fluorescence of the mixture was measured using a FP8000 fluorescence spectrometer (JASCO, Tokyo, Japan). Excitation of NBD-DMPE at 460 nm induces fluorescence emission at 530 and 588 nm, corresponding to the emissions from NBD-DMPE and rho-DMPE, respectively. Dilution of rho-DMPE owing to membrane fusion with NBD-DMPE increases the fluorescence intensity at 530 nm and decreases the intensity at 588 nm. The fusion efficiency was evaluated using calibration samples with known fluorescence^23^,^26^, which allowed us to assess membrane fusion following the freeze–thaw cycles based on the lipid composition of the liposomes. The FRET dissolution efficiency of the exosome–liposome mixture was defined as *E*_*FD*_ = *F*_530_/(*F*_530_ + *F*_588_), where *F*_530_ and *F*_588_ represent the fluorescent intensities at 530 and 588 nm, respectively. We prepared a calibration curve of liposomes containing 0.650, 0.500, 0.250, 0.185, 0.125, and 0.063 mol% of NBD-DMPE and rho-DMPE. These concentrations represent the lipid dilution ratio of 0.65, 1, 2, 3, 4, and 5, respectively. The *E*_*FD*_ values were obtained for DOPC, DOPS and DOTAP liposomes containing the indicated concentrations of NBD-DMPE and rho-DMPE, and were plotted against the lipid dilution ratio. The lipid dilution ratio of the labelled liposome was then calculated from the standard curve.

### Flow cytometry

#### Exosome bead coupling and flow cytometry

Exosomes (6 μg, protein) were incubated with 2 μL of aldehyde/sulfate latex beads (4 μm diameter) for 15 min at room temperature. The volume was made up to 1 mL using MEMS buffer and the mixture was incubated overnight on a rotary shaker at 4 °C. To block remaining binding sites, the exosome-coated beads were incubated for 30 min with 92 mM glycine at room temperature. After three washes in stain buffer, the presence of CD63 protein on the membrane surface of exosome bound to the beads was examined by single-color immunofluorescence labelling with PE-conjugated anti-CD63 monoclonal antibody. As isotype controls, exosome-coated beads were stained with PE-conjugated rat IgG2a after incubation for 1 h at room temperature. All data were collected on a LSR Fortessa cell analyser (BD Bioscience), and analysed with FlowJo software (Treestar, Inc., San Carlos, CA, USA).

#### Cellular uptake

Purified Raw 264.7-derived exosomes (300 μg/mL, protein) were fused with 100 μM of liposomes (1:1 by volume) using the freeze–thaw method described above and labelled with 62.8 μM CFSE for 30 min at 37 °C, and the volume was made up to 100 μL with PBS buffer. The labelled exosome suspension was transferred to a PD Spin Trap G-25 to remove free CFSE. The resulting Raw 264.7 exosome–liposome hybrids or Raw 264.7 exosomes (4.5 μg protein in exosome) were incubated with 1 × 10^5^ HeLa cells for 4 h at 37 °C. The cells were then washed twice with PBS, detached using trypsin, and resuspended in stain buffer. The fluorescence intensity was measured on the LSR Fortessa cell analyser.

### Confocal laser scanning microscopy

The cellular uptake of exosomes into HeLa cells was visualized by confocal laser scanning microscope equipped with plan-APOCHROMAT 40× oil immersion objective (LSM780, Carl Zeiss, German) with excitation by Argon laser at 488 nm. CFSE-labelled Raw 264.7 exosome–liposome hybrids or CFSE-labelled Raw 264.7 exosomes (3 μg protein in exosomes) were incubated with 1 × 10^5^ HeLa cells for 4 h at 37 °C under 5% CO_2_. The cells were washed twice with PBS, the medium was replaced with fresh medium, and the cells were cultured for 4 h at 37 °C under 5% CO_2_.

## Additional Information

**How to cite this article**: Sato, Y. T. *et al.* Engineering hybrid exosomes by membrane fusion with liposomes. *Sci. Rep.* **6**, 21933; doi: 10.1038/srep21933 (2016).

## Supplementary Material

Supplementary Information

## Figures and Tables

**Figure 1:**
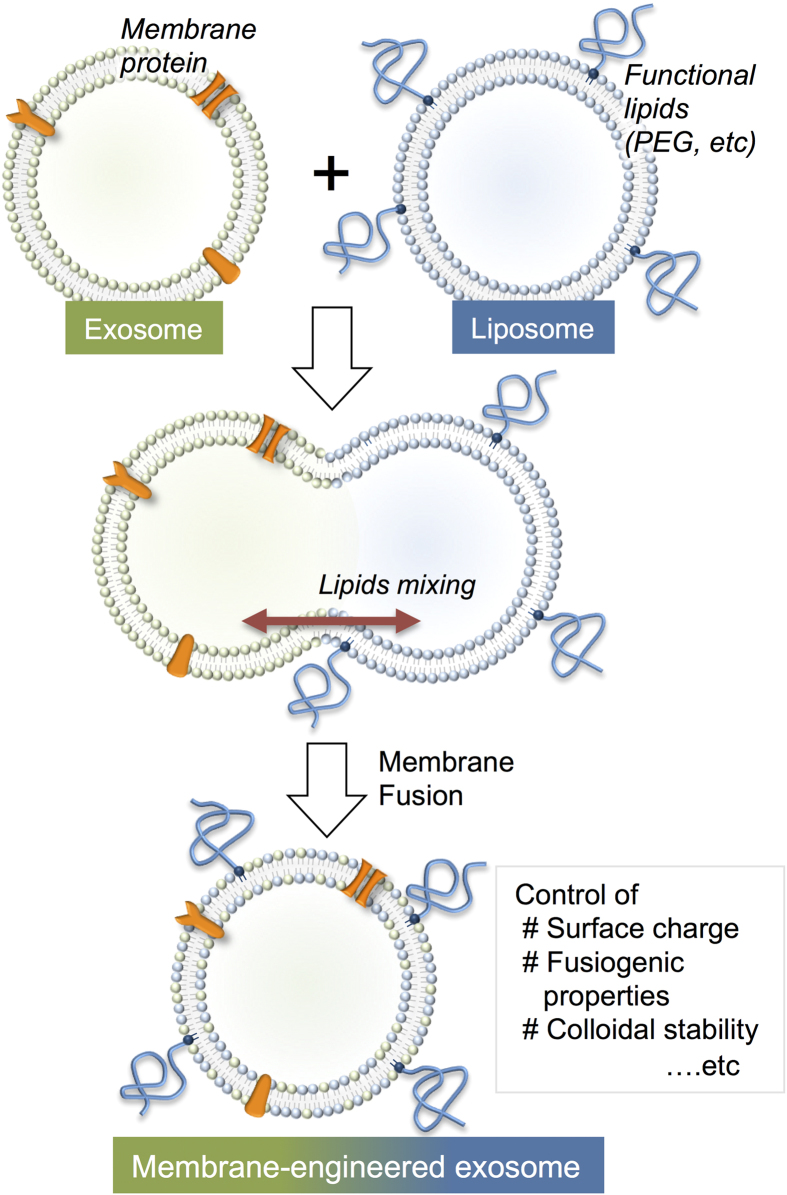
Schematic of method used to engineer the exosome–liposome hybrids.

**Table 1:** Fusion efficiencies between Raw 264.7 cell-derived exosomes and liposome after freeze–thaw cycles.

Exosome	Phospholipid	Lipid dilution ratio/-
−	DOPC	2.1 ± 0.1
−	DOPS	1.7 ± 0.0
−	DOTAP	4.8 ± 0.1
−	DOPS/PEG-DSPE	2.9 ± 0.1
+	DOPC	3.3 ± 0.2
+	DOPS	5.3 ± 0.2
+	DOTAP	5.2 ± 0.4
+	DOPS/PEG-DSPE	7.6 ± 0.5

**Table 2:** Fusion efficiencies between of the CMS7-HE cell-derived exosomes and liposome after freeze–thaw cycles.

Exosome	Phospholipid	Lipid dilution ratio/-
+	DOPC	3.5 ± 0.2
+	DOPS	8.0 ± 0.2
+	DOTAP	9.3 ± 0.8
