# Supplementary material for: Engineering hybrid exosomes by membrane fusion with liposomes
Source: Sci Rep. 2016 Feb 25;6:21933. doi: 10.1038/srep21933 (PMC4766490; doi:10.1038/srep21933)
Supplement: Supplementary Information [file srep21933-s1.pdf]

Supplementary Information

Engineering hybrid exosomes by membrane fusion with liposomes

*Yuko. T. Sato[1], Kaori Umezaki[1], Shinichi Sawada[1,2], Sada-atsu Mukai[1,2], Yoshihiro Sasaki[2], Naozumi Harada[3], Hiroshi Shiku[3] and Kazunari Akiyoshi[1,2,]**

[1]Japan Science and Technology Agency (JST), The Exploratory Research for Advanced Technology (ERATO), Bio-nanotransporter Project, Katsura Int'tech Center, Katsura, Nishikyo-ku, Kyoto 615-8530, Japan

[2]Department of Polymer Chemistry, Graduate School of Engineering, Kyoto University, Katsura, Nishikyo-ku, Kyoto, 615-8510, Japan

[3] Department of Immuno-Gene Therapy, Graduate School of Medicine, Mie University, Tsu 514-8507, Japan

Supplementary Table S1

Table S1. Average size of Raw 264.7 exosomes or exosome-liposome hybrids after freeze-thawing. The diameters were evaluated by nanoparticle tracking analysis.

Exosome	Phospholipid	Size / nm
+	-	150 ± 100
+	DOPC	230 ± 170
+	DOPS	250 ± 200
+	DOTAP	210 ± 110
+	DOPS/PEG-DSPE	190 ± 80

Supplementary Figures S1-S4

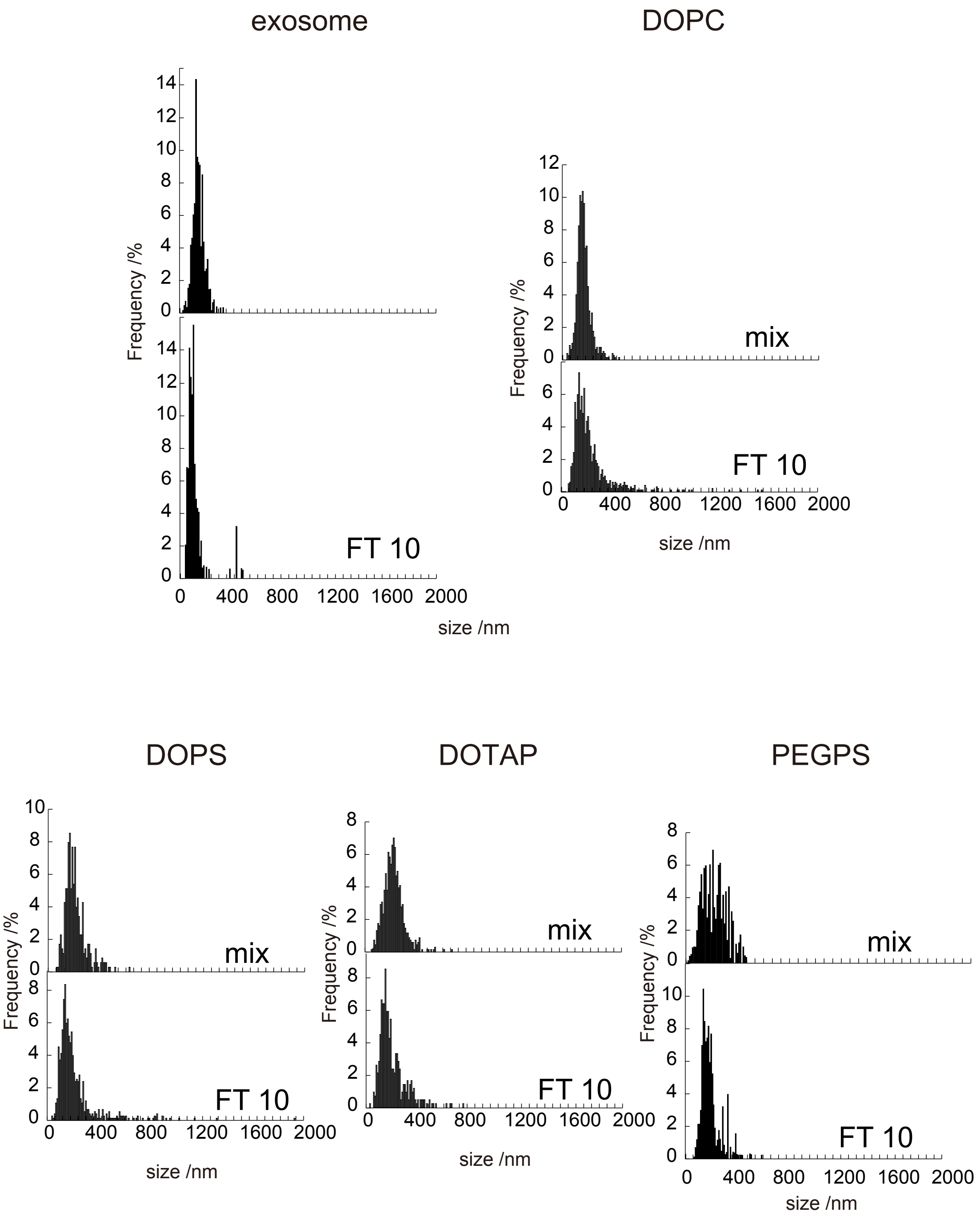

Figure S1 | Size distribution of exosome or exosome-liposome mixture before (mix) and after freeze-thawing (FT10) measured by nano particle tracking assay.

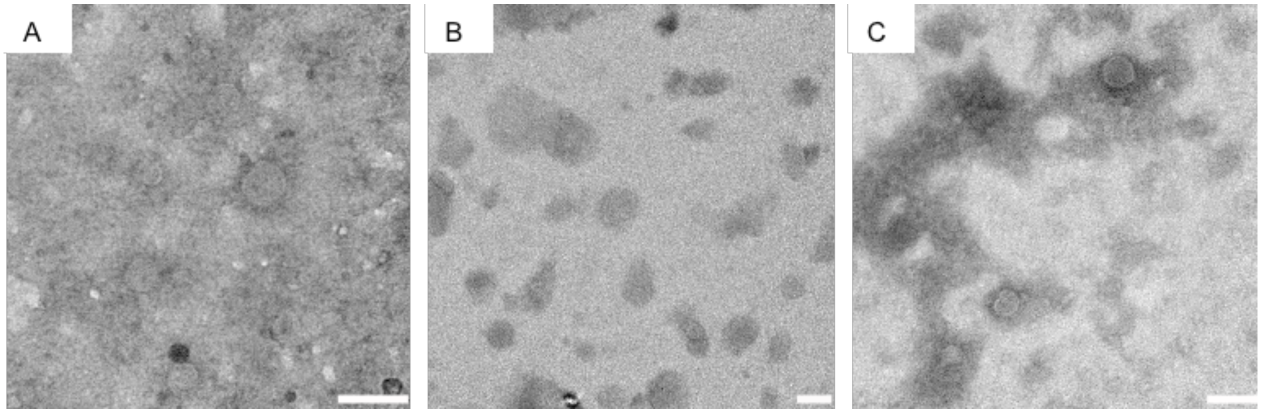

Figure S2 | Exosome as observed by transmission electron microscopy. A) exosomes, B) DOPS liposomes, C) exosomes-DOPS hybrid prepared by freeze-thawing. Scale bars represent 100 nm. Exosomes were placed on 100-mesh formvar/carbon coated copper grids pretreated with 1% alcian blue for 3 min. After rinsing with distilled water, exosomes in 2% paraformaldehyde (PFA) were dropped on grids and air-dried. Samples were stained with 2.5% phosphotungstic acid and observed by a HT7700-Transmission electron microscope (Hitachi, Japan) at an accelerating voltage of 100 kV.

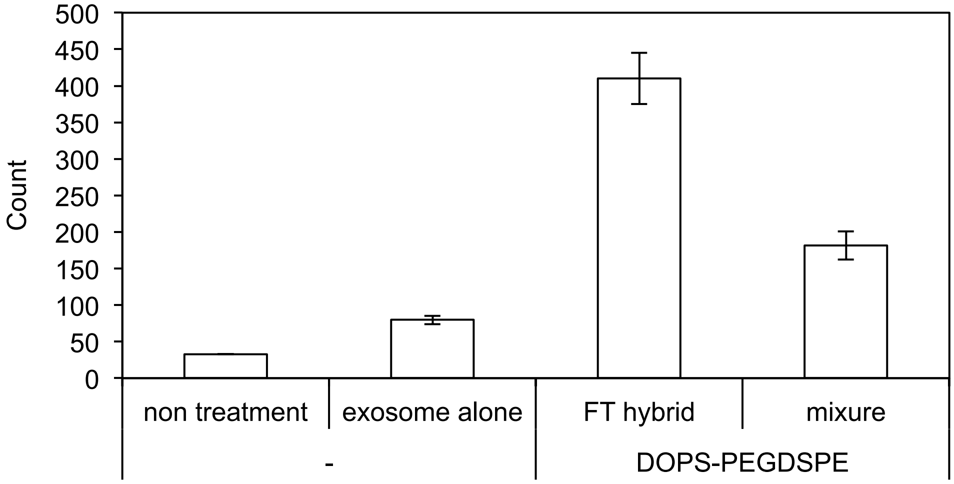

Figure S3 | Effect of freeze-thawing on the cellular uptake efficiency. The efficiencies were evaluated by mean fluorescence intensities of cells determined by flow cytometry with or without treatment of freeze-thawing to the mixture of exosome and DOPS/PEGDSPE liposome. Statistical significance was evaluated by t-test from 3 times of FACS analysis ($p<0.01$).

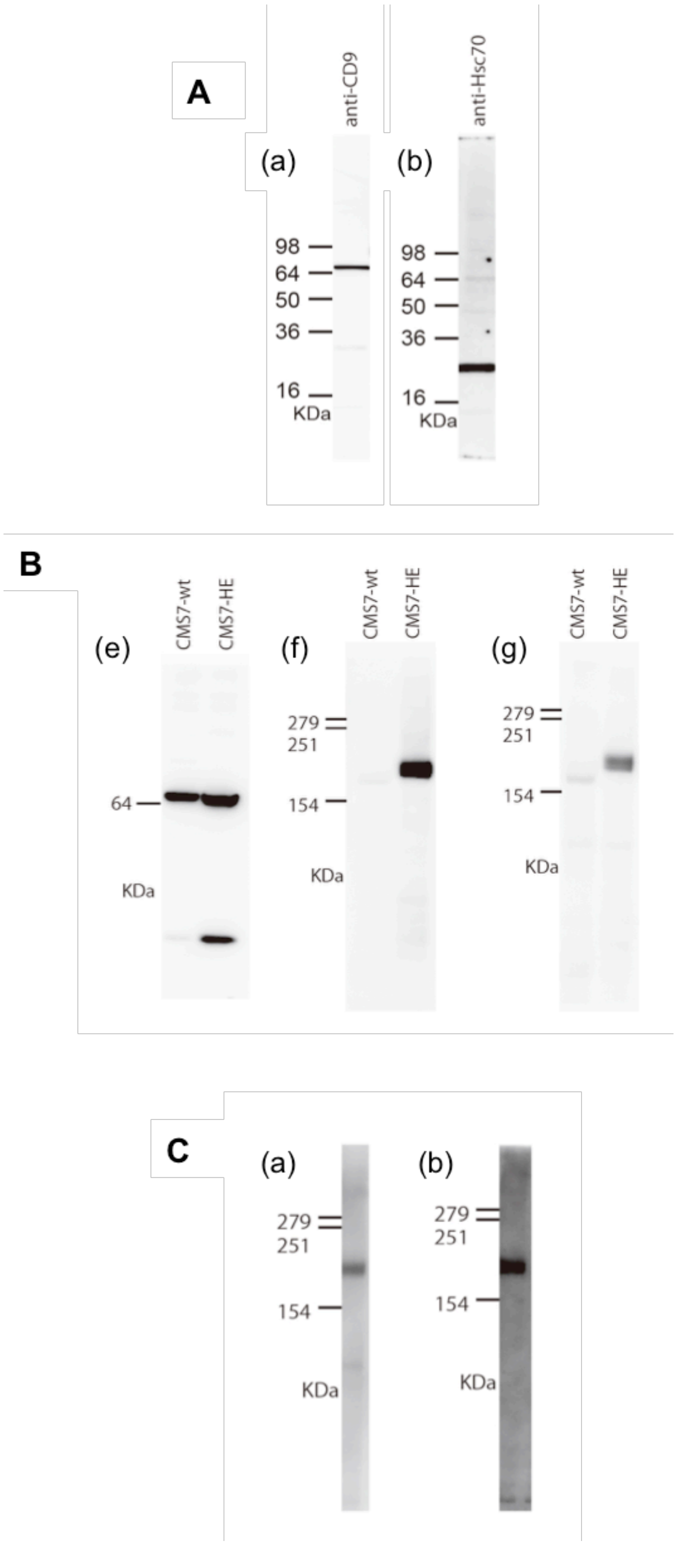

Figure S4 | Full length of image of western blotting for Figure 2 a, b (A), Figure 4 e, f, g (B) and Figure 5 (C).
